# Preventing Fractures in Diabetic Dialysis Patients

**DOI:** 10.5812/ijem.5384

**Published:** 2012-09-30

**Authors:** E. Michael Lewiecki

**Affiliations:** 1 New Mexico Clinical Research & Osteoporosis Center, Albuquerque, New Mexico, USA

**Keywords:** Osteoporosis, Treatment, Chronic Kidney Disease, Diabetes Mellitus

Dear Editor,

Skeletal health is a concern for patients with type 2 diabetes mellitus. There is an increased risk of fracture associated with diabetes (1) and some of the medications (e.g., thiazolidinediones) used to treat the diabetes (2). Chronic kidney disease (CKD), which may occur as a complication of diabetes or independently, is also a risk factor for fracture (3). The mechanisms linking diabetes, thiazolidinediones, and CKD with fractures are complex and not fully understood; however, the clinical challenges associated with the management of these disorders are ever-present and in need of solutions (4).

O Saito et al. addressed one aspect of this conundrum in a study of the skeletal effects of raloxifene in a small study (N = 60) of postmenopausal Japanese women on maintenance hemodialysis (5). Study subjects were in two groups, with and without type 2 diabetes; women in each group were randomized into subgroups receiving raloxifene 60 mg daily or no raloxifene for one year. The aim of the study was to compare changes in bone turnover markers and speed of sound (SOS) measured by heel quantitative ultrasound (QUS) in subjects receiving or not receiving raloxifene. In women with postmenopausal osteoporosis, a decrease in bone turnover markers with antiresorptive therapy (i.e. raloxifene) is associated with improvement in BMD and a reduction in fracture risk. SOS, which can be used to estimate bone mineral density and T-score, is directly correlated with bone strength and fracture risk when measured by validated QUS instruments.

At the end of one year in the Saito et al. study, the diabetics receiving raloxifene had a significantly higher SOS than the control diabetics (P = 0.02), but otherwise there were no significant differences in SOS or bone turnover markers. Comparing the change from baseline values between the subgroups, it was found that SOS increased and serum N-telopeptide (NTX) decreased in diabetics and non-diabetics receiving raloxifene compared with the control subgroups (P < 0.05).

The findings of this study suggest the possibility that raloxifene may have beneficial skeletal effects in postmenopausal women on hemodialysis. However, this is a hypothesis that requires further study before raloxifene can be recommended for clinical use in these patients. At this time, there are more questions than answers. In patients with end-stage CKD, it is not clear whether a reduction in bone turnover markers or an increase SOS with treatment is associated with a reduction in fracture risk. Since some postmenopausal women with end-stage CKD could have CKD-mineral and bone disorder with low bone turnover, an antiresorptive medication may not be appropriate for all. There are safety concerns with the use of raloxifene, a drug associated with increased risk of thromboembolic events, in patients with end-stage CKD and/or diabetes mellitus, since these patients are at high baseline risk for cardiovascular disease. More data are needed to better define the bone disease in patients with end-stage CKD, with or without diabetes, and determine whether pharmacological interventions can reduce fracture risk with a favorable balance of benefit and risk.
